# Multibacillary Leprosy Presenting as Anetoderma in a Young Teenager

**DOI:** 10.1155/2020/8847310

**Published:** 2020-07-29

**Authors:** Niraj Parajuli, Shraddha Shrestha, Laila Lama, Rushma Shrestha, Sumida Tiwari, Anupama Karki

**Affiliations:** ^1^Department of Dermatology & Venereology, National Academy of Medical Sciences, Bir Hospital, Kathmandu, Nepal; ^2^Department of Pathology, National Academy of Medical Sciences, Bir Hospital, Kathmandu, Nepal

## Abstract

Anetoderma presents as a circumscribed area of slack skin. It can present as either primary or secondary, if associated with other conditions. Leprosy is one of the causes of secondary anetoderma, but it is not commonly reported, especially in multibacillary leprosy. Here, we report a case of a 16-year-old young girl who presented with fever, joint pain, and only three anetodermic plaques. A slit skin smear from the lesion showed multiple acid-fast bacilli with a bacillary index of 3+, thus confirming the diagnosis of leprosy. This case is unique since multibacillary leprosy presented with only few anetoderma lesions in a young teenager girl from a leprosy-eliminated country.

## 1. Introduction

Anetoderma refers to a circumscribed area of slack skin associated with a loss of dermal substance on palpation and a loss of elastic tissue on histological examination [1]. Anetoderma is an elastolytic disorder characterized by localized areas of flaccid skin, which may be depressed, macular, or papular [2]. “Primary” anetoderma is associated with no localized underlying cutaneous disease, whereas “secondary” anetoderma can be attributed to some associated condition like leprosy, tuberculosis, urticaria pigmentosa, pityriasis versicolor, granuloma annulare, and others [1].

Here, we report a case of multibacillary leprosy presenting as anetoderma in a young teenager girl during the post-elimination era.

## 2. Case Report

A 16-year-old female from the Terai region presented to the emergency department with complaints of high-grade fever for 5 days. She was being managed empirically with parenteral antibiotics. An opinion was sought from the dermatological team regarding few asymptomatic, skin-colored lesions over the extremities. The soft plaques were first noticed over the left lower leg and, then, over the right arm within a period of 6 months. There was no significant past and family history.

**Figure 1:**
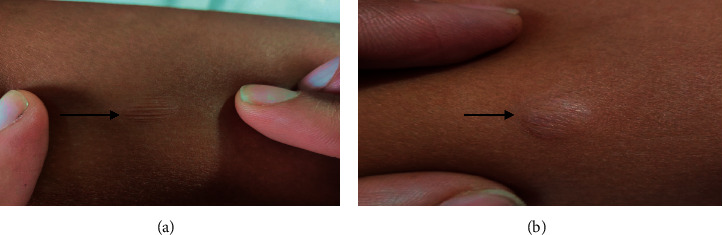
(a) Skin-colored atrophic plaques which became flat on stretching. (b) the skin becoming lax after release, returning to the initial wrinkly texture.

**Figure 2:**
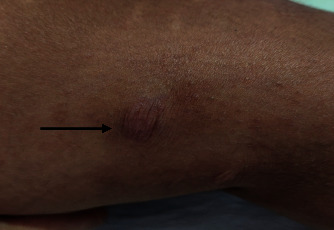
Atrophic wrinkled plaques over the left lower thigh.

**Figure 3:**
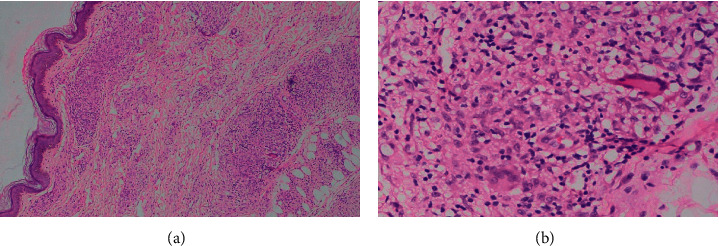
(a) Histopathological examination showing dermal granuloma with epithelioid cell aggregates and few giant cells (H&E stain 40x magnification). (b) Histopathological examination in higher magnification showing epitheloid cell granulomas with few multinucleated giant cells (H&E stain 100x magnification).

On general examination, an ill-looking young female with fever, bilateral pedal pitting edema, and diffuse swelling of the face was observed. Vital signs were all within normal limits. On skin examination, few round-to-oval plaques with an atrophic, wrinkled surface of approximate 1 × 1 cm^2^ were present over the right arm, forearm, and left lower leg. On stretching, atrophic plaques became flat (Figure 1(a)), and on leaving the skin lax, the plaques returned to initial texture (Figure 1(b)).Similar plaques were also present over the left lower thigh (Figure 2). All the plaques had decreased sensation to cold and touch. Bilateral ulnar nerves and the left common peroneal nerve were enlarged and tender. No motor deficit or deformity was noted during the examination. A slit skin smear with Ziehl–Neelsen stain was performed, revealing multiple acid-fast bacilli with a bacillary index (BI) of 3+. Excisional biopsy and histopathological examination of atrophic plaques from the right arm revealed multiple well-formed granulomas consisting of epithelioid cells with peripheral rimming of lymphocytes in the upper dermis and perineural and periadnexal lymphocytic infiltrates along with few multinucleated giant cells (Figures 3(a) and 3(b)).

Venereal disease research laboratory (VDRL) tests and serologies for HIV and hepatitis B and C were negative. The antinuclear antibodies test was negative as well.

The patient was diagnosed as a case of multibacillary leprosy with Type 1 reaction. Treatment was started on multidrug therapy-multibacillary type (MDT-MB) along with oral prednisolone 40 mg/day on a tapering dose. The patient and the parents were counselled regarding the nature of the disease and were advised for regular follow-up.

Contact examination in immediate family members did not show any features of leprosy.

## 3. Discussion

Anetoderma, first described by Jadassohn, is characterized by localized areas of loss of substance and elastic tissue with flaccid skin and often leads to a herniation phenomenon [3]. Usually, it presents as crops of round or oval pink macules or plaques 0.5–1 cm in diameter seen mostly over the trunk, thighs, and upper arms [1]. The skin lesion fades and flattens from the center outwards, leaving wrinkled, atrophic macules which yield on pressure [4].

Primary anetoderma occurs idiopathically from normal skin and is strongly associated with antiphospholipid syndrome. Secondary anetoderma is reported with tuberculosis, leprosy, lupus, syphilis, granuloma annulare, B- and T-cell lymphomas, and other conditions [5]. The exact etiology of anetoderma is unknown, but the condition is associated with an increase in elastolysis [6]. The loss of elastic fibers can be due to several factors including a decrease in elastolytic enzyme inhibitors, increased production of elastolytic enzymes, or autoimmune-mediated destruction of fibers [7].

Special staining methods such as Verhoeff-Van Gieson are required for the confirmatory diagnosis of the condition, which identify the elastic fibers, elastolysis, and elastorrhexis that affect both the papillary and reticular dermis [6]. This staining was not performed due to lack of its availability in our institute.

There are a few case reports of leprosy presenting as secondary anetoderma. Anetoderma is a rare form of presentation of Hansen's disease [6, 7].

This case is being reported due to the unusual and rare presentation of leprosy. Our patient presented with a multibacillary type of leprosy but with only few atrophic wrinkled plaques with a BI of 3+ and without any family history of leprosy. This case suggests that there is still ongoing transmission in the community. Nepal has been able to maintain its status of leprosy elimination till date, but this rare form of leprosy in the community poses a big threat to this status due to the delay in diagnosis. Leprosy presents with varied morphology, so people from endemic areas should be screened for leprosy even on a slightest of suspicion. Active case finding should be intensified to find the hidden cases for early detection and treatment. This case also emphasizes the role of more strong leprosy programs to provide early diagnosis and prompt treatment, as well as the role of clinician for prompt diagnosis of the rare forms of leprosy. Physicians should suspect and rule out leprosy in any unusual dermatological presentations despite the leprosy elimination status of the country.

## Data Availability

All the data used to support the findings of this study are included within the article.
